# Prevalence of and Factors Associated With Substance Use Among Canadian Medical Students

**DOI:** 10.1001/jamanetworkopen.2021.33994

**Published:** 2021-11-17

**Authors:** Anees Bahji, Marlon Danilewitz, Eva Guerin, Brandon Maser, Erica Frank

**Affiliations:** 1 Cumming School of Medicine, Department of Psychiatry, University of Calgary, Calgary, Alberta, Canada; 2 The British Columbia Centre on Substance Use, Vancouver, British Columbia, Canada; 3 Department of Psychiatry, University of Toronto, Toronto, Ontario, Canada; 4 Department of Psychology, Carleton University, Ottawa, Ontario, Canada; 5 Institute of Health Policy, Management, and Evaluation, University of Toronto, Toronto, Ontario, Canada; 6 The University of British Columbia Faculty of Medicine, Vancouver, British Columbia, Canada; 7 The Annenberg Physician Training Program in Addiction Medicine, Bethel, Minnesota

## Abstract

This cross-sectional study investigates the national prevalence of tobacco, alcohol, cannabis, and nonmedical prescription stimulant use among Canadian medical students.

## Introduction

Substance use is prevalent in medical students and physicians and has important implications for their personal health, training, and clinical practice.^1,2,3,4^ However, there is a lack of recent, rigorous studies of medical student substance use from representative samples.^4^ This study aimed to identify the national prevalence of tobacco, alcohol, cannabis, and nonmedical prescription stimulant (NPS) use among Canadian medical students. Furthermore, we explored whether substance use varied by medical student demographics and was associated with psychological morbidity.

## Methods

This cross-sectional study was conducted using an electronic survey of medical students across all 17 Canadian medical schools. Reporting followed the Strengthening the Reporting of Observational Studies in Epidemiology (STROBE) reporting guideline. The University of British Columbia institutional review board approved this study, and participants supplied written informed consent. Detailed methods have been previously published^5^ and are included the eAppendix in the Supplement.

We purposively sampled all Canadian medical students from all years of study: a target population of 11 469. We recruited participants during 2 preplanned waves: November 30 to December 14, 2015 (n = 2981), and February 14 to March 1, 2016 (n = 1457). We analyzed data for the current study between February and July 2021. Primary outcomes included the prevalence of alcohol, tobacco, cannabis, and NPS use. We conducted logistic regression models and computed odds ratios (ORs) using R statistical software version 3.5.3 (R Project for Statistical Computing). Significance was set at *P* < .05, using 2-sided χ^2^ tests to detect significant associations.

## Results

We analyzed 4438 valid surveys from the 11 469 students who received the survey, giving a participation rate of 40.2%. Participant characteristics have been previously described.^5^ Among 4438 respondents, 2857 (64.3%) were female; the mean (SD) age was 24.2 (3.5) years. The lifetime self-reported prevalence rates were 45.6% (95% CI, 44.0%-47.2%) for cannabis, 8.3% (95% CI, 7.4%-9.2%) for NPS, and 6.8% (95% CI, 5.9%-7.7%) for cigarettes; past-month excessive alcohol use was 46.4% (95% CI, 44.8%-48.1%) (Table 1). In multivariable logistic regression, we found that male sex was associated with greater self-reported prevalence rates of all 4 substances (excessive alcohol: OR, 1.66; 95% CI, 1.44-1.91; current cigarette use: OR, 2.31; 95% CI, 1.71-3.11; cannabis in past 12 months: OR, 1.41; 95% CI, 1.19-1.69; NPS in past 12 months: OR, 1.72; 95% CI, 1.22-2.42). Lower age was associated with self-reported cannabis use in the past 12 months (OR, 0.95; 95% CI, 0.93-0.97) and excessive alcohol use (OR, 0.96; 95% CI, 0.94-0.98), whereas older students were more likely to self-report cigarette use (OR, 1.17; 95% CI, 1.14-1.21) (Table 2).

**Table 1.** Weighted Prevalence of Alcohol Use, Tobacco Use, Cannabis Use, and NPS Use by Gender

Substance use measure	Medical students, No. (%) [95% CI]			*P* value
	Total sample (n = 4438)	Female (n = 2441)	Male (n = 1997)	
Alcohol use				<.001
None	734 (16.5) [15.3-17.8]	387 (15.9) [14.4-17.3]	347 (17.4) [15.3-19.5]	.24
Non-excessive	1642 (37.0) [35.4-38.6]	1060 (43.4) [41.4-45.4]	583 (29.2) [26.7-31.7]	<.001
Excessive	2061 (46.4) [44.8-48.1]	994 (40.7) [38.8-42.7]	1067 (53.4) [50.8-56.1]	<.001
Smoking history				<.001
Never	4136 (93.2) [92.3-94.1]	2332 (95.6) [94.7-96.4]	1804 (90.3) [88.7-92.0]	<.001
Past	205 (4.6) [3.9-5.3]	74 (3.0) [2.4-3.7]	130 (6.5) [5.2-7.9]	<.001
Current	97 (2.2) [1.7-2.7]	34 (1.4) [0.9-1.9]	63 (3.2) [2.2-4.1]	<.001
Cannabis use				<.001
Never used	2414 (54.4) [52.8-56.0]	1390 (56.9) [55.0-58.9]	1024 (51.3) [48.6-54.0]	<.001
Used				
But not in the past 12 mo	1105 (24.9) [23.5-26.3]	611 (25.0) [23.3-26.8]	495 (24.8) [22.5-27.1]	.87
But not in the past 30 d	568 (12.8) [11.7-13.9]	291 (11.9) [10.7-13.2]	277 (13.9) [12.0-15.6]	.08
Used in the past 30 d	351 (7.9) [7.0-8.8]	149 (6.1) [5.2-7.0]	202 (10.1) [8.5-11.7]	<.001
NPS				<.001
Never used	4069 (91.7) [90.8-92.6]	2277 (93.3) [92.3-94.3]	1791 (89.7) [88.1-91.3]	<.001
Used				
But not in the past 12 mo	198 (4.5) [3.7-5.2]	89 (3.6) [2.8-4.4]	109 (5.5) [4.2-6.7]	.01
But not in the past 30 d	81 (1.8) [1.4-2.3]	29 (1.2) [0.8-1.6]	53 (2.7) [1.8-3.5]	<.001
Used in the past 30 d	90 (2.0) [1.6-2.3]	46 (1.9) [1.4-2.4]	44 (2.2) [1.5-3.5]	.49
Alcohol use, mean (SD) [95% CI]				
Days drinking any alcohol, per month	5.1 (0.1) [4.9-5.3]	4.6 (0.1) [4.4-4.8]	5.7 (0.2) [5.4-6.0]	<.001
Days binge drinking, per month	1.1 (0.0) [1.0-1.1]	0.8 (0.0) [0.7-0.8]	1.5 (0.1) [1.4-1.6]	<.001
Tobacco use, mean (SD) [95% CI]				
Days smoking in past month among current smokers	8.1 (1.0) [6.1-10.1]	8.4 (1.7) [5.2-11.6]	7.9 (1.3) [5.3-10.4]	.80
Cigarettes per day among current smokers	2.1 (0.3) [1.5-2.7]	1.6 (0.5) [0.7-2.6]	2.3 (0.4) [1.6-3.1]	.28

Abbreviation: NPS, nonmedical use of prescription stimulants.

**Table 2.** Results of Multivariable Logistic Regression of Factors Associated With Use of Alcohol, Tobacco, Cannabis, and NPS

Independent variable	Dependent variable							
	Excessive alcohol		Current cigarette use		Cannabis in past 12 mo		NPS in past 12 mo	
	OR (95% CI)	*P* value	OR (95% CI)	*P* value	OR (95% CI)	*P* value	OR (95% CI)	*P* value
Demographics								
Male (vs female)	1.66 (1.44-1.91)	<.001	2.31 (1.71-3.11)	<.001	1.41 (1.19-1.69)	<.001	1.72 (1.22-2.42)	<.001
Age	0.96 (0.94-0.98)	<.001	1.17 (1.14-1.21)	<.001	0.95 (0.93-0.97)	<.001	0.99 (0.93-1.05)	.69
Year of study	0.98 (0.92-1.05)	.66	1.03 (0.90-1.17)	.70	1.04 (0.96-1.13)	.30	0.97 (0.82-1.15)	.75
Campus (DME sv main)	1.14 (0.92-1.41)	.25	0.67 (0.44-1.02)	.06	1.24 (0.94-1.63)	.13	1.05 (0.73-1.51)	.79
Has a regular physician	1.09 (0.94-1.26)	.27	0.93 (0.68-1.27)	.63	0.85 (0.72-1.02)	.08	1.07 (0.74-1.54)	.73
Mental illness								
Mood disorder	0.81 (0.61-1.06)	.13	0.62 (0.37-1.03)	.06	0.80 (0.58-1.11)	.19	0.64 (0.35-1.16)	.14
Anxiety disorder	0.93 (0.72-1.20)	.57	1.31 (0.80-2.16)	.28	1.17 (0.86-1.58)	.33	0.91 (0.50-1.65)	.76
K6 summary	1.02 (1.01-1.05)	.03	1.06 (1.01-1.11)	.01	1.01 (0.98-1.04)	.50	1.08 (1.03-1.14)	<.001
Mental well-being								
Resilience (CD-RISC2)	1.13 (1.06-1.20)	<.001	1.25 (1.11-1.41)	<.001	1.10 (1.03-1.18)	.01	1.18 (1.02-1.37)	.02
Flourishing mental health (MHC-SF)	1.00 (0.87-1.15)	.98	1.03 (0.76-1.38)	.85	0.95 (0.80-1.12)	.53	0.97 (0.69-1.37)	.88
Burnout								
Emotional exhaustion	0.81 (0.55-1.18)	.27	1.30 (0.64-2.62)	.47	0.96 (0.61-1.52)	.86	0.54 (0.26-1.16)	.11
Depersonalization	0.98 (0.76-1.25)	.85	1.45 (0.90-2.33)	.13	1.20 (0.89-1.63)	.23	0.97 (0.57-1.65)	.91
Overall burnout	1.19 (0.79-1.79)	.41	0.88 (0.40-1.93)	.75	1.02 (0.62-1.69)	.94	2.99 (1.29-6.92)	.01

Abbreviations: CD-RISC2, Connor-Davidson Resilience Scale 2-item; K6, Kessler Psychological Distress Scale 6-item; MHC-SF, Mental Health Continuum–Short Form; NPS, nonmedical use of prescription stimulants; OR, odds ratio.

There was no significant association between self-reported substance use and mood or anxiety disorders. However, students with higher psychological distress were more likely to report excessive alcohol use (OR, 1.02; 95% CI, 1.01-1.05), current cigarette use (OR, 1.06; 95% CI, 1.01-1.11), or NPS use (OR, 1.08; 95% CI, 1.03-1.14) use in the past 12 months. Students with greater resilience were also more likely to report all 4 categories of substance use. Meeting the criteria for burnout was significantly associated with NPS use (OR, 2.99; 95% CI, 1.29-6.92) but not with other substances. We found associations between NPS use and decreased ability to handle workloads due to physical issues (OR, 0.31; 95% CI, 0.18-0.54) and mental health issues (OR, 0.46; 95% CI, 0.30-0.71).

## Discussion

To our knowledge, this study is one of the largest and most comprehensive studies of medical student substance use.^4^ Canadian medical students reported similar rates of alcohol use, higher rates of cannabis use, and lower rates of tobacco and NPS use than Canadian postsecondary students.^6^ These findings matter because medical students' substance use may indicate their ability to cope with stress and their risk of burnout, and their patient counseling practices on substance use.^1,2^ For example, NPS use was associated with greater psychological distress and burnout and lower coping ability. However, higher resilience scores were unexpectedly associated with more substance use for all 4 substances queried, which warrants further study. Study limitations include the cross-sectional study design, potential response bias, the age of the data (2015-2016), the omission of alternative tobacco forms, and a uniform cutoff for binge drinking for men and women (≥5 drinks). In addition, future research should examine the association between other demographic variables, such as race and ethnicity, with substance use, explore the mechanisms and mediating factors for the observed associations, examine the prevalence of substance use disorders (in addition to use), and investigate the effects of interventions to reduce substance-related harms among medical students.

## Notes

Supplement. **eAppendix.** Supplemental Methods
