# Supplementary material for: Prevalence of and Factors Associated With Substance Use Among Canadian Medical Students
Source: JAMA Netw Open. 2021 Nov 17;4(11):e2133994. doi: 10.1001/jamanetworkopen.2021.33994 (PMC8600384; doi:10.1001/jamanetworkopen.2021.33994)
Supplement: Supplement. — eAppendix. Supplemental Methods [file jamanetwopen-e2133994-s001.pdf]

Supplemental Online Content

Bahji A, Danilewitz M, Guerin E, Maser B, Frank E. Prevalence of and factors associated with substance use among Canadian medical students. *JAMA Netw Open.* 2021;4(11):e2133994. doi:10.1001/jamanetworkopen.2021.33994

eAppendix. Supplemental Methods

This supplemental material has been provided by the authors to give readers additional information about their work.

© 2021 Bahji A et al. *JAMA Network Open.*

eAppendix: Supplementary Methods

Sample

This was a cross-sectional study that employed a self-administered, anonymous, electronic survey of medical students in all years of study at all 17 Canadian medical schools: Dalhousie University Faculty of Medicine, McGill University Faculty of Medicine, Michael G. DeGroote School of Medicine at McMaster University, Memorial University of Newfoundland Faculty of Medicine, Northern Ontario School of Medicine, Queen's University School of Medicine, Faculté de médicine de l'Université de Montréal, Faculté de médecine et des sciences de la santé de l'Université de Sherbrooke, Faculté de médecine de l'Université Laval, University of Alberta Faculty of Medicine and Dentistry, University of British Columbia Faculty of Medicine, Cumming School of Medicine at University of Calgary, Max Rady College of Medicine at University of Manitoba, University of Ottawa Faculty of Medicine, University of Saskatchewan College of Medicine, University of Toronto Faculty of Medicine, and Schulich School of Medicine & Dentistry at Western University.

We used purposive sampling to conduct a total-population sample of Canadian medical students, with a target population of 11,469.[1]

Survey procedure

Ethical approval was obtained through the University of British Columbia Behavioural Research Ethics Board (reference number H14-02774; approved on October 1, 2015). The survey was developed and administered using the web-based survey platform SurveyMonkey (SurveyMonkey Inc., San Mateo, California). The survey instrument and instructions were provided in English and French. The questionnaire was piloted by a focus group comprised of 25 individuals from both English- and French-speaking backgrounds to ensure ease of use and validity of the online survey format.

We invited participants to complete the survey during two separate 2-week recruitment periods (November 30-December 14, 2015, and February 14-March 1, 2016). Links to the survey were distributed to medical students via email listservs previously established for the national medical student representative bodies (the Canadian Federation of Medical Students and the Fédération médicale étudiante du Québec). Medical students who contributed to the creation and/or piloting of the survey, as well as students who were on leave from medical school for ≥ 4 weeks were asked not to participate. Invitation emails were sent out at the outset of each recruitment period, with a single follow up email at the 1-week mark. We conducted an additional 2-week recruitment period (March 4th-March 18th, 2016), with two additional invitation emails, at two schools that were identified as having the lowest response rates (>1 standard deviation below the mean school response rate).

All responses were voluntary and anonymous. Survey participation was incentivized using three concurrent strategies: 1) Participants had the option to enter a draw for one of two iPads ($1000 value each) after exiting the survey; 2) the school with the highest per capita response rate received a prize of $1000 per year of study ($4000 total), earmarked for each class' graduation

© 2021 Bahji A et al. *JAMA Network Open.*

fund; and 3) participants had the option to vote from a set of charitable organizations for donation of a subset of grant funds ($2000), with funds being distributed proportional to votes.

Study measures

Personal characteristics. The survey included items about demographics, including age, gender, year of study, relationship status, medical school, site of study (i.e., main campus or distributed medical education site), specialty of interest, and whether one had a regular medical doctor for their personal care. Students' clinical/pre-clinical training status was derived from the date of curriculum transition at each of the 17 medical faculties, respondents' year of study, and the date of survey response. For specialty of interest, respondents were asked "Choose the one specialty you are now *most* interested in pursuing." Specialties were grouped for analyses according to Supplemental Table 1 (presented at the end of these supplemental methods). For querying personal physician use, respondents were asked "Do you have a regular medical doctor for your personal care?"

Substance use. Self-report frequency of marijuana use and the non-medical use of prescription stimulants (NPS) (e.g., Ritalin, Dexedrine or Adderall) were queried by asking about the use of these substances outside of a doctor's orders, using questions adapted from McCabe et al. 2005.[2] Respondents were asked "How often, if ever, have you used any of the drugs listed below? Do not include anything you used under a doctor's orders." Responses were categorized into four categories: (1) never used; (2) used, but not in the past 12 months; (3) used, but not in the past 30 days; or (4) used in the past 30 days.

To determine alcohol use, students were asked how many days in the past month they had any alcoholic beverages (defined as 1 can/bottle of beer, 1 glass of wine, 1 can/bottle of wine cooler, 1 cocktail, or 1 shot of liquor) and how many days they binge drank (defined as 5 or more drinks on one occasion[3]) using validated questions adapted from the Centers for Disease Control and Prevention (CDC) Behavioral Risk Factor Surveillance System (BRFSS).[4,5] Specifically, respondents were asked the following: "During the past month, on about how many days did you drink any alcoholic beverages?"; "On the days when you drank, about how many drinks did you drink, on average? (A drink is 1 can/bottle of beer or wine cooler, 1 glass of wine, 1 cocktail, or 1 shot of liquor)"; and "How often in the past month did you have ≥5 drinks on one occasion?"

Scoring of alcohol consumption was based on criteria from the National Institute on Alcohol Abuse and Alcoholism.[3,6] Alcohol consumption was classified as ***'excessive'*** in the previous month if respondents met at least one of the following criteria: 1) reported at least one occasion on which they consumed five or more drinks (i.e., reported one or more episodes of binge drinking); or 2) *men* who drank more than *two* drinks per day on average or *women* who drank more than *one* drink per day on average. Alcohol consumption was classified as ***'non-excessive'*** for respondents that consumed alcohol in the past month but did not meet criteria for "excessive" alcohol use. Alcohol consumption was classified as ***'none'*** for respondents that reported zero alcohol consumption in the past month.

Cigarette use questions were also adapted from the CDC-BRFSS.[4,5] Respondents were asked the following questions: "Have you smoked at least 100 cigarettes in your entire life?"; "Do you *now* smoke cigarettes every day, some days, or not at all?"; "During the past 30 days, on the days you

© 2021 Bahji A et al. *JAMA Network Open.*

smoked, how many cigarettes did you smoke per day?"; and "On how many of the past 30 days did you smoke part or all of a cigarette?" Respondents were categorized into three categories for smoking history (never, past, or current) and three categories for current smoking status (ex-smoker, some days, or every day). The mean and standard deviation were also calculated for days spent smoking in the past month and cigarettes per day amongst those that reported current cigarette use.

Mental illness. This study included four distinct measures of mental illness: diagnosed mood disorders, diagnosed anxiety disorders, suicidal ideation, and psychological distress. Questions regarding mental health diagnoses and suicidal ideation were taken verbatim from the Canadian Community Health Survey - Mental Health 2012 (CCHS-MH) questionnaire.[7-9] The CCHS-MH was a nationally representative community mental health survey that was conducted by Statistics Canada (Government of Canada) between January to December 2012.[7-9] Items from the CCHS-MH were adapted from a telephone interview survey script into an online, self-administered format. It targeted the household population aged 15 or older in the 10 Canadian provinces. Persons living on reserves and other Aboriginal settlements, full-time members of the Canadian Armed Forces, and the institutionalized population were excluded from the CCHS-MH. Computer-assisted telephone and in-person interviews were conducted, and no proxy interviews were permitted for the CCHS-MH. More information on the CCHS-MH is available elsewhere.[10]

Respondents were prompted before each mood and anxiety disorder question to "only indicate long-term conditions that are expected to last or have already lasted six months or more and that have been diagnosed by a health professional." For mood and anxiety disorders, respondents were asked, "Do you have a mood disorder such as depression, bipolar disorder, mania, or dysthymia?" and "Do you have an anxiety disorder such as a phobia, obsessive-compulsive disorder, or a panic disorder?"[7]

For lifetime and past 12-months suicidal ideation, respondents were asked "Have you ever…" or "In the past 12 months, have you seriously thought about committing suicide or taking your own life?" Suicidal ideation questions, adapted from the CCHS-MH, were initially sourced from the World Mental Health-Composite International Diagnostic Interview (WMH-CIDI) 3.0.[11]

Psychological distress. For this report, we considered having a diagnosed mood or anxiety disorder or self-reported suicidal ideation as evidence of overt mental illness. However, many individuals with symptomatic, undiagnosed mental illness would not be captured by these measures. Therefore, we also used a well-validated inventory for nonspecific psychological distress—the Kessler Psychological Distress Scale 6-item (K6)—as a screening tool to capture both diagnosed and undiagnosed serious mental illness.[12-15] Nonspecific psychological distress, a measure of broadly defined emotional problems, has been widely used in multiple scales (such as the K6) and surveys as a screening measure for serious mental illness.[12-15]

The K6 is designed to yield a global measure of psychological distress based on questions related to depressive and anxiety symptoms experienced in the past month or year.[12,14,15] It has been used to screen for serious mental illness in national health surveys around the world, including in the United States and Australian national health surveys, the World Health Organization WMH-CIDI, and the CCHS-MH.[9,12-15] K6 responses were scored using unweighted summative scoring

© 2021 Bahji A et al. *JAMA Network Open.*

of Likert responses, according to established methods.[13,15,16] In the present analysis, we coded psychological distress as a dichotomous categorical variable as K6 score ≥ 9 (i.e., moderate or severe psychological distress) versus < 9 (mild or no distress). There is no universal clinical standard for scoring the K6 because score cut-offs are based on population-specific validation.[15] Therefore, we chose a K6 score cut-off of ≥9 based on prior methods[17] and informed by previously identified stratum-specific likelihood ratios for past 1-month and past 12-month depression in Canadian validation data.[16] For a K6 score ≥ 9, the sensitivity and specificity for predicting major depression in the past 1 month are 0.70 and 0.94, respectively.[16]

Mental well-being. Medical student resilience was assessed using the Connor-Davidson Resilience Scale 2-item (CD-RISC2).[18-20] This instrument was used with the express consent and under the appropriate licensing agreement with the copyright holders of the CD-RISC2.[20] The CD-RISC2 was scored from 0-8 by unweighted summation of Likert responses, as previously described.[18,20] For regression analyses, CD-RISC2 scores were left as a continuous independent variable, with higher scores indicating better resilience.

Positive mental health was assessed using the Mental Health Continuum-Short Form (MHC-SF).[21-24] The MHC-SF is a previously-validated instrument comprised of 14 questions that assess aspects of emotional, psychological, and social well-being. It classifies respondents' mental health as flourishing (high positive emotions, high positive functioning), languishing (low positive emotions, low positive functioning), or moderate (neither flourishing nor languishing).[21-24] The MHC-SF has been validated across multiple populations,[24] including in college students,[22] and its outcome measure of 'floursihing' mental health has been shown to be protective against all-cause mortality,[23] mental illness,[25,26] suicidal behaviour,[22] and academic impairment.[22] The instrument was scored as a categorical variable, according to established methods,[24] to categorize respondents into the following categories: '***flourishing***', '***moderately mentally healthy***', or '***languishing'***. For regression analyses, respondents were grouped into '***flourishing'*** versus '***not flourishing***' (i.e., moderately mentally health and languishing).

Burnout. Medical student burnout was queried using a two-item adapted and validated version of the Maslach Burnout Inventory (MBI).[27-32] The MBI was used with express consent and under the appropriate licensing agreement with the copyright holders of the MBI.[32] The MBI is the reference standard for burnout assessment and has been validated in multiple populations, including medical professionals at all levels of training.[27-32] As previously described and validated in medical professionals and trainees,[27-31] we measured burnout using two single-item measures of emotional exhaustion ("How often do you feel burned out from your schooling/work?") and depersonalization ("How often do you feel you've become more callous toward people since you started schooling/work?"), adapted from the full MBI. Responses ranged from 0 ('never') to 6 ('every day'), with anchors at 1 ('a few times a year or less'), 2 ('once a month or less'), 3 ('a few times a month'), 4 ('once a week') and 5 ('a few times a week'). These items have been shown to correlate strongly with the emotional exhaustion and depersonalization domains of burnout as measured by the full MBI in a sample of over 10,000 medical students, residents, and practicing physicians.[29] Furthermore, these items show strong associations with multiple published outcomes in medical professionals, such as suicidality, professionalism, and medical errors.[28] The area under the receiver-operating characteristic curve for the emotional exhaustion and depersonalization single items against their respective full MBI

© 2021 Bahji A et al. *JAMA Network Open.*

domain measure is 0.94 and 0.93.[28] The positive predictive values of the single-item thresholds for high levels of emotional exhaustion and depersonalization are 88.2% and 89.6%, with positive likelihood ratios of 14.9 and 23.4, respectively.[28,29]

Respondents' scores were manipulated according to the procedures established in previous validation studies.[28,29] Specifically, a high score on either the emotional exhaustion or depersonalization subscales were defined as a response of 'at least weekly' in the respective item, in accordance with previously defined cut-offs.[28,29] Emotional exhaustion and depersonalization were included in regression analyses as dichotomous categorical variables, using this cut-off of 'at least weekly'. Overall burnout, also included in regression analyses as a dichotomous categorical variable, was defined from the single-item measures by the presence of high levels of emotional exhaustion ***and/or*** depersonalization, as has been described in prior literature.[27-31,33]

Learner mistreatment. Learner mistreatment was queried using 10 separate questions, asking respondents whether they had been harassed or belittled (never, some, or severely) during medical school by the following different groups: 1) other students, 2) residents or fellows, 3) pre-clinical professors, 4) clinical professors/attending physicians, and 5) patients. Questions were taken verbatim from a prior study of U.S. medical students.[34] We scored responses using unweighted summative scoring, as described previously.[34] Specifically, we collapsed the responses of 'some' and 'severely' into one category, with both 'some' or 'severely' responses receiving a score of 1 for each belittlement/harassment group (i.e., other students, residents or fellows, pre-clinical professors, clinical professors, and patients) and a response of 'never' receiving a score of 0. Scores were summed across all five of the different groups for both belittlement and harassment to give belittlement and harassment continuous scores of 0-5.

Perceived medical school support. Respondents were asked about their medical school's personal health promotion environment using questions described previously.[35] Prior results from this study have shown strong correlations between students' and Deans' perceptions of their respective schools' health promotion environments.[35] Responses were scored according to a five-point Likert scale, from from 'strongly disagree' (0) to 'strongly agree' (4).

Career satisfaction. Career satisfaction was queried by asking "If you re-lived your life, would you still want to become a physician?" Responses were scored according to a five-point Likert scale: 'definitely not' (0), 'probably not' (1), 'maybe' (2), 'probably' (3), and 'definitely' (4).

Work impact. To assess the impact of respondents' mental and physical health on their functioning as medical students, respondents were asked "In the past 4 weeks, how often did your *physical health [or mental health]* health make it difficult for you to handle your workload as a medical student?" Responses were scored according to a Likert scale, from 'all of the time' (0) to 'none of the time' (4).

Patient counseling attitudes and practices. Questions regarding patient counseling attitudes and practices were taken verbatim from a prior instrument implemented in U.S. medical students.[36] To query students' patient counseling attitudes and practices regarding alcohol use and smoking cessation, respondents were asked "How relevant do you think this will be in your

© 2021 Bahji A et al. *JAMA Network Open.*

intended practice: 1) Smoking cessation (among smokers); and 2) Alcohol?"; and "With a typical general medicine patient, how often do you actually talk about: 1) Smoking cessation (among smokers); and 2) Alcohol?" For counseling relevance questions, responses were scored according to a three-point Likert scale, from 'not at all' (0) to 'highly' (2). For questions regarding actual counseling practices, respondents who had not yet interacted with patients (i.e., pre-clinical/pre-clerkship students) were asked to respond 'N/A' (not applicable) (n = 2779 [unweighted]; n=2375 [weighted])). Valid responses were scored according to a three-point Likert scale, from 'never/rarely' (0) to 'usually/always' (2).

Data Analysis

We analyzed data using RStudio version 3.5.3.[37] Potential differences between nonrespondents and respondents were assessed with a twofold approach. First, we compared results between late-respondents (those who responded in the last two weeks of recruitment) and early-respondents (those who responded in the first two weeks of recruitment). This is a well-accepted method for evaluating nonresponse bias in surveys with multiple waves of recruitment, by using late-respondents as proxies for nonrespondents.[38,39] Second, we weighted data for nonresponse using the raking ratio method[40] to match students' demographic characteristics (gender, year of study, medical school, and school of study) with national enrollment data.[1] Analyses were run using both weighted and unweighted data and there were no significant differences between weighted and unweighted results. Therefore, only results from weighted data are presented. Weighted results are based on male and female respondents in years 1–4, as national enrollment demographic data were only available for these subgroups.[1]

For comparisons between early- and late-respondents, late-respondents (mean age = 25.9 years, SD = 3.5) were significantly older than early-respondents (mean age = 23.9 years, SD = 3.4, $P < .001$). Early-respondents also reported more recent NPS (i.e., 'used, but not in the past 30 days') than late-respondents (2.1% vs 0.0%, $P < .001$). Apart from these findings, there were no significant differences between early- and late-respondents on any of the other variables assessed.

Results were also compared between respondents from the entire first (November–December 2015) and second (February–March 2016) response periods to determine whether the timing of response was associated with differing outcomes. Participants in the second response period were significantly older than those in the first response period ($P < .001$). There was also a significant difference in year of study between these two response periods ($P < .001$), with second-year students being more likely to have responded in the first response period and fourth-year students being more likely to have responded in the second response period. There was also a significant difference in those reporting mood disorders between these two response periods ($P < .001$), with those with a diagnosed mood disorder being more likely to have responded in the second response period. However, there were no significant differences between these two response periods for the remainder of variables assessed.

Associations between categorical variables were evaluated using χ^2 tests of association. Post hoc pairwise comparisons were conducted with a Bonferroni correction. We used generalized linear modelling to examine differences in alcohol and tobacco use continuous variables between different groups. We used multivariable logistic regression to identify associations with

© 2021 Bahji A et al. *JAMA Network Open.*

categorical substance use outcome variables, as well as with categorical outcome variables for professional attitudes and behaviours. Regression analyses were completed using weighted data. Predictor variables were included using forced entry, with odds ratios adjusted for the entirety of the predictor set. Participants with no valid data (i.e., missing across all variables of interest) as well as those with missing data for the key demographic variables of age, gender, year of study, and medical school were not included in weighted analyses because these variables were required for weighting.

Supplemental Table 1. Specialty of Interest Classification.

Specialty of Interest (n)	Classification for analyses
Anaesthesia (129)	Lifestyle
Dermatology (63)	
Diagnostic Radiology (96)	
Emergency Medicine (318)	
Ophthalmology (63)	
Family Medicine (1242)	Primary Care
Preventative Medicine and Public Health (28)	
Cardiac Surgery (27)	Surgical
General Surgery (144)	
Neurosurgery 48	
Obstetrics/Gynecology (187)	
Orthopedic Surgery (104)	
Otolaryngology (59)	
Plastic Surgery (63)	
Urology (50)	
Vascular Surgery (25)	
Internal Medicine (674)	Internal Medicine
Neurology (90)	
Neurology-Pediatric (13)	Paediatrics
Pediatrics (348)	
Psychiatry (176)	Psychiatry
Physical Med & Rehab (40)	Other
Anatomical Pathology (24)	
Medical Biochemistry (1)	
General Pathology (10)	
Hematological Pathology (7)	
Laboratory Medicine (6)	
Nuclear Medicine (1)	
Medical Genetics (11)	
Medical Microbiology (13)	
Neuropathology (1)	
Radiation Oncology (20)	
Undecided (330)	Undecided

© 2021 Bahji A et al. *JAMA Network Open.*

References

1. Association of the Faculties of Medicine of Canada. Canadian Medical Education Statistics 2016. 2016. https://afmc.ca/sites/default/files/CMES2016-reduced.pdf. Accessed 2019 Jan 12. Accessed January 12, 2019.
2. McCabe SE, Knight JR, Teter CJ, Wechsler H. Non-medical use of prescription stimulants among US college students: prevalence and correlates from a national survey. *Addiction.* 2005;99:96-106.
3. National Institute on Alcohol Abuse and Alcoholism. National Institutes of Health. https://www.niaaa.nih.gov/alcohol-health/overview-alcohol-consumption/moderate-binge-drinking. Published 2019. Accessed.
4. Nelson DE, Holtzman D, Bolen J, Stanwyck CA, Mack KA. Reliability and validity of measures from the Behavioral Risk Factor Surveillance System (BRFSS). *Soz Praventivmed.* 2001;46 Suppl 1:S3-42.
5. Centers for Disease Control and Prevention. BRFSS Questionnaires. U.S. Department of Health & Human Services. https://www.cdc.gov/brfss/questionnaires/index.htm. Published 2019. Accessed.
6. Frank E, Elon L, Naimi T, Brewer R. Alcohol consumption and alcohol counselling behaviour among US medical students: cohort study. *British Medical Journal.* 2008;337.
7. Statistics Canada. Canadian Community Health Survey - Mental Health (CCHS). http://www23.statcan.gc.ca/imdb/p2SV.pl?Function=getSurvey&SDDS=5015. Updated September 10, 2013. Accessed August 13, 2014.
8. Statistics Canada. Canadian Community Health Survey (CCHS) – Mental Health: Questionnaire. In: Stastics Canada, ed2011.
9. Statistics Canada. Canadian Community Health Survey (CCHS) – Mental Health: User Guide Microdata Files. In: Statistics Canada Health Statistics Division, ed2013.
10. Statistics Canada. Table 105-1101 Mental health profile, Canadian Community Health Survey - Mental Health (CCHS), by age group and sex, Canada and provinces. http://www5.statcan.gc.ca/cansim/a26?lang=eng&retrLang=eng&id=1051101&tabMode=dataTable&p1=1&p2=-1&srchLan=-1&pattern=mental+health+profile. Published 2013. Updated September 17, 2013. Accessed.
11. Kessler RC, Ustun TB. The World Mental Health (WMH) Survey Initiative Version of the World Health Organization (WHO) Composite International Diagnostic Interview (CIDI). *Int J Methods Psychiatr Res.* 2004;13(2):93-121.
12. Kessler RC, Andrews G, Colpe LJ, et al. Short screening scales to monitor population prevalences and trends in non-specific psychological distress. *Psychol Med.* 2002;32(6):959-976.
13. Furukawa TA, Kessler RC, Slade T, Andrews G. The performance of the K6 and K10 screening scales for psychological distress in the Australian National Survey of Mental Health and Well-Being. *Psychol Med.* 2003;33(2):357-362.
14. Kessler RC, Barker PR, Colpe LJ, et al. Screening for serious mental illness in the general population. *Arch Gen Psychiatry.* 2003;60(2):184-189.
15. Kessler RC, Green JG, Gruber MJ, et al. Screening for serious mental illness in the general population with the K6 screening scale: results from the WHO World Mental Health (WMH) survey initiative. *Int J Methods Psychiatr Res.* 2010;19 Suppl 1:4-22.

© 2021 Bahji A et al. *JAMA Network Open.*

16. Cairney J, Veldhuizen S, Wade TJ, Kurdyak P, Streiner DL. Evaluation of 2 measures of psychological distress as screeners for depression in the general population. *Canadian Journal of Psychiatry-Revue Canadienne De Psychiatrie.* 2007;52(2):111-120.
17. Maser B, Danilewitz M, Guérin E, Findlay L, Frank E. Medical Student Psychological Distress and Mental Illness Relative to the General Population: A Canadian Cross-Sectional Survey. *Academic Medicine.* 2019;94(11):1781-1791.
18. Vaishnavi S, Connor K, Davidson JR. An abbreviated version of the Connor-Davidson Resilience Scale (CD-RISC), the CD-RISC2: psychometric properties and applications in psychopharmacological trials. *Psychiatry Res.* 2007;152(2-3):293-297.
19. Connor KM, Davidson JR. Development of a new resilience scale: the Connor-Davidson Resilience Scale (CD-RISC). *Depress Anxiety.* 2003;18(2):76-82.
20. Davidson JRT, Connor KM. Connor-Davidson Resilience Scale Manual. *Personal communication from the authors.* Unpublished 2014.
21. Keyes CL. The mental health continuum: from languishing to flourishing in life. *J Health Soc Behav.* 2002;43(2):207-222.
22. Keyes CL, Eisenberg D, Perry GS, Dube SR, Kroenke K, Dhingra SS. The relationship of level of positive mental health with current mental disorders in predicting suicidal behavior and academic impairment in college students. *J Am Coll Health.* 2012;60(2):126-133.
23. Keyes CLM, Simoes EJ. To Flourish or Not: Positive Mental Health and All-Cause Mortality. *American Journal of Public Health.* 2012;102(11):2164-2172.
24. Keyes CLM. Brief description of the mental health continuum short form (MHC-SF). https://www.aacu.org/sites/default/files/MHC-SFEnglish.pdf. Published 2009. Accessed.
25. Grant F, Guille C, Sen S. Well-being and the risk of depression under stress. *PLoS One.* 2013;8(7):e67395.
26. Keyes CL, Dhingra SS, Simoes EJ. Change in level of positive mental health as a predictor of future risk of mental illness. *Am J Public Health.* 2010;100(12):2366-2371.
27. Dyrbye LN, West CP, Satele D, et al. Burnout among U.S. medical students, residents, and early career physicians relative to the general U.S. population. *Acad Med.* 2014;89(3):443-451.
28. West CP, Dyrbye LN, Satele DV, Sloan JA, Shanafelt TD. Concurrent validity of single-item measures of emotional exhaustion and depersonalization in burnout assessment. *J Gen Intern Med.* 2012;27(11):1445-1452.
29. West CP, Dyrbye LN, Sloan JA, Shanafelt TD. Single item measures of emotional exhaustion and depersonalization are useful for assessing burnout in medical professionals. *J Gen Intern Med.* 2009;24(12):1318-1321.
30. West CP, Shanafelt TD, Kolars JC. Quality of life, burnout, educational debt, and medical knowledge among internal medicine residents. *JAMA.* 2011;306(9):952-960.
31. Shanafelt TD, Boone S, Tan L, et al. Burnout and satisfaction with work-life balance among US physicians relative to the general US population. *Arch Intern Med.* 2012;172(18):1377-1385.
32. Maslach C, S. J, Leiter M. *Maslach Burnout Inventory Manual. 3rd ed.* Palo Alto, CA: Consulting Psychologists Press; 1996.
33. Thomas NK. Resident burnout. *JAMA.* 2004;292(23):2880-2889.

© 2021 Bahji A et al. *JAMA Network Open.*

34. Frank E, Carrera JS, Stratton T, Bickel J, Nora LM. Experiences of belittlement and harassment and their correlates among medical students in the United States: longitudinal survey. *BMJ.* 2006;333(7570):682.
35. Frank E, Hedgecock J, Elon LK. Personal health promotion at US medical schools: a quantitative study and qualitative description of deans' and students' perceptions. *BMC Med Educ.* 2004;4(1):29.
36. Frank E, Carrera JS, Elon L, Hertzberg VS. Predictors of US medical students' prevention counseling practices. *Prev Med.* 2007;44(1):76-81.
37. RStudio Team. Integrated Development for R. The R Foundation for Statistical Computing. http://www.rstudio.com/. Published 2019. Accessed.
38. Compton MT, Frank E. Mental health concerns among Canadian physicians: results from the 2007-2008 Canadian Physician Health Study. *Compr Psychiatry.* 2011;52(5):542-547.
39. Phillips AW, Reddy S, Durning SJ. Improving response rates and evaluating nonresponse bias in surveys: AMEE Guide No. 102. *Med Teach.* 2016;38(3):217-228.
40. Battaglia M, Hoaglin D, Frankel M. Practical considerations in raking survey data. *Survey Practice.* 2009;2(5). https://surveypractice.scholasticahq.com/article/2953-practical-considerations-in-raking-survey-data.

© 2021 Bahji A et al. *JAMA Network Open.*

DRAFT ' DO NOT DISTRIBUTE

CFMS'FMEQ National Medical Student Health and Well'Being Survey

Brandon Maser[1]; Dr. Marlon Danilewitz, M.D.[2]; Dr. Bryce Durafourt, M.D.[3], Dr. Erica Frank, MD, MPH[4]

1. MD Candidate, Class of 2016, Queen's University 2.Psychiatry PGY1, University of British Columbia Faculty of Medicine 3.Neurology PGY1, Queen's University Faculty of Medicine 4.Tier 1 Canada Research Chair in Preventive Medicine and Population Health, Professor at University of British Columbia's School of Population and Public Health.

Objectives

1. To collect baseline data on basic mental and physical health of Canadian medical students;
2. To better understand the contributing factors to poor and to good medical student physical and mental health, including colleague behaviours, system characteristics, and environmental stressors and supports;
3. To explore utilization of student mental and physical health services and potential barriers to access;
4. To assess any correlation between students' health and their attitudes regarding patient care and professional satisfaction.

Survey Items:

Key to comparable items:

CCHS G survey items used from the Canadian Community Health Survey (CCHS) – Mental Health Questionnaire – November 30, 2011

AMS G survey items used with permission from Dr. Erica Frank's (UBC) survey of American medical students

CPHS G CMA's Canadian Physician Health Survey

NPS G CMA's National Physician Survey

Australian G Australia's Beyond Blue National Mental Health Survey of Doctors and Medical Students

CAIR G Canadian Association of Interns and Residents national survey

These notations will not appear on the final questionnaire, but have been left in for now to assist our collaborators and the Behavioral Ethics Review Board with information about why we chose some of the questions and the wording we chose. The default is always to make exact comparability with the CMA Canadian Physician Health questionnaire and American Medical student questionnaires (marked CPHS and AMS, respectively), and other people in the Canadian population (items marked CCHS). Language was altered only where necessary, and only to improve clarity, to alter the format to a written survey (in the case of the CCHS), and to make the items applicable for a medical student population.

Express consent and appropriate licensing agreements with those responsible for intellectual property of the Maslach Burnout Inventory and ConnorGDavidson Resilience Scale have been obtained.

Participant Information and Consent:

© 2021 Bahji A et al. *JAMA Network Open.*

To start the survey, students will be presented with our letter of invitation (see below). It is based on the UBC Behavioral REB approved letter for use with the Canadian Medical Association questionnaire (surveying Canadian physicians), previously submitted by Dr. Erica Frank.

Dear Colleague,

As a physician4in4training, you know that your own health is subject to factors and forces similar to those that impact your patients, and that you are not immune to stressors on your mental and physical health. Therefore, the Canadian Federation of Medical Students (CFMS) and the Fédération Médicale Étudiante du Québec (FMEQ) are collecting new information on the health and behaviors of medical students studying undergraduate medicine at institutions across Canada, so that we can develop and advocate for programs to improve our health.

The total approximate time for completion of this survey is 15 minutes.

Please be assured that your response to this survey is purely voluntary. Your confidentiality will be respected. No direct identifying data will be collected from participants. Demographic data with the potential for identifying students will be collected anonymously (IP addresses will not be stored) and data will be kept confidential by the research investigators. Research records may be inspected in the presence of the Investigator or his or her designate by representatives of Canadian Medical Foundation and the UBC Research Ethics Board for the purpose of monitoring the research.

All data, including demographic data, will only be presented at the aggregate level, with no identification of or reference to individual participants in published and presented reports. Data stratified by medical school may be presented and/or distributed in the instance that it will assist specific schools in improving medical student health programs and resources. In this instance, student data may be presented on the aggregate level based on year of study and school. However, information regarding sensitive data, such as substance use, mental health diagnoses, and suicidal ideation, will be excluded from data presented at the level of individual classes.

Your participation, including your answers to any individual items, is completely voluntary (but greatly appreciated); you are free to answer all, some, or none of the questions without prejudice to their student status or class standing. By your participation, your consent is implied.

This online survey is hosted by a web4survey company based out of the United States of America, and as such, is subject to U.S. laws. In particular, the U.S. Patriot Act allows authorities access to the records of internet service providers. This survey or questionnaire does not ask for personal identifiers or any information that may be used to identify you. The websurvey company servers record incoming IP addresses of the computer that you use to access the survey but no connection is made between your data and your computer's IP address. If you choose to participate in the survey, you understand that your

responses to the survey questions will be stored and may be accessed in the U.S. The security and privacy policy for the websurvey company can be found at the following link: https://www.surveymonkey.com/mp/policy/security

We realize that some of the questions in the survey are personal in nature and deal with sensitive subject matter. Should you feel distress at any point during the survey, please feel free to discontinue your participation, and do not hesitate to visit ***http://suicideprevention.ca/thinkingCaboutCsuicide*** *for information on a crisis centre in your province.*

For more information on the range of physician health services offered to you by the CMA and Divisions, including access to confidential support services, please contact the physician health program in your province/territory or the CMA Centre for Physician Health and Well4being at 148774CMA444YOU (148774 26244968). A full list of physician health programs and contact numbers is available at https://www.cma.ca/En/Pages/provincial4physician4health4programs.aspx

If you have questions about the survey, please contact the Principal Investigator, Dr. Erica Frank, M.D., M.P.H., at erica.frank@ubc.ca; or the co4principal investigator, Brandon Maser, B.Sc., M.D. Candidate, at bmaser@qmed.ca.

Who can you contact if you have complaints or concerns about the study?
If you have any concerns or complaints about your rights as a research participant and/or your experiences while participating in this study, contact the Research Participant Complaint Line in the UBCOffice of Research Ethics at 604482248598 or if long distance e4mail RSIL@ors.ubc.ca or call toll free 14 877482248598.

In advance, thank you for your time and attention in this very important project. On behalf of the CFMS and FMEQ and the medical students of Canada who will benefit from your participation in this survey, thank you.

To promote quality and comparability, most of these questions are taken verbatim (with permission) from other surveys of large and relevant comparator populations, especially Canadian physicians, U.S. medical students, and other Canadian young adults.

SURVEY

Demographics:

1) What is your age? [CCHS AN3_Q03]

2) *The CFMS and FMEQ identify that gender is a spectrum, and are gender sensitive and inclusive organizations. We are asking about gender for demographic purposes and to uncover risk and protective factors related to medical student health and well4being.*

With what gender* do you identify?

- Female
- Male
- Other (please specify)

3) Do you consider yourself to be...? [CCHS SDC_Q14]

- Heterosexual
- Homosexual
- Bisexual
- Other

4) Relationship Status (pick one) [AMS q6]

- Single, never married
- Member of an unmarried couple
- Married/Commonlaw
- Separated/Divorced
- Widowed

5) Are you an Aboriginal person that is, First Nations, Métis or Inuit? First Nations includes Status and NonGStatus Indians [CCHS SDC_Q05].

G Yes

G No

6) What University and campus do you attend for medical school?

- Dalhousie University G Halifax, NS
- Dalhousie University G Saint John, NB
- McGill University
- McMaster University G St. Catherines, ON
- McMaster University G Kitchener, ON
- McMaster University G Hamilton, ON
- Memorial University of Newfoundland
- Northern Ontario School of Medicine G Thunder Bay, ON
- Northern Ontario School of Medicine G Sudbury, ON
- Queen's University
- University of Alberta

- University of British Columbia G Vancouver, BC
- University of British Columbia G Victoria, BC
- University of British Columbia G Prince George, BC
- University of British Columbia G Kelowna, BC
- University of Calgary
- Université Laval
- University of Manitoba
- Université de Montréal G Montréal, QC
- Université de Montréal G Mauricie, QC
- University of Ottawa
- Université de Sherbrooke G Chicoutimi, QC
- Université de Sherbrooke G Moncton, NB
- Université de Sherbrooke G Sherbrooke, QC
- University of Toronto G St. George Campus
- University of Toronto G Mississauga, ON
- University of Saskatchewan G Regina, SK
- University of Saskatchewan G Saskatoon, SK
- Western University G London, ON
- Western University G Windsor, ON

7) In what year of your medical program are you currently?
    - Pre6med
    - First
    - Second
    - Third
    - Fourth
    - Fifth
    - Other (please specify)

8) Choose the one speciality you are now most interested in pursuing: [question from AMS q10, options from Canadian Residency Matching Service listed specialties for application]
    - Anatomical Pathology
    - Anesthesia
    - Cardiac Surgery
    - Dermatology
    - Diagnostic Radiology
    - Emergency Medicine
    - Family Medicine
    - General Pathology

- General Surgery
- Hem. Pathology
- Internal Medicine
- Laboratory Medicine
- Medical Biochemistry
- Medical Genetics
- Medical Microbiology
- Neurology
- Neurology – Pediatric
- Neuropathology
- Neurosurgery
- Nuclear Medicine
- Obstetrics/Gynecology
- Ophthalmology
- Orthopedic Surgery
- Otolaryngology
- Pediatrics
- Physical Med & Rehab
- Plastic Surgery
- Psychiatry
- Preventative Medicine and Public Health
- Radiation Oncology
- Urology
- Vascular Surgery
- Other (please specify)
- Undecided

9) What certificates, diplomas, or degrees have you previously obtained (Check ALL that apply): [Adapted from 2010 NPS Student edition]

None	
Trades certificate or diploma	
Other non-university certificate or diploma (including Cégep in Québec)	
University certificate or diploma, below Bachelor's degree level	
Bachelor's degree(s)	
University certificate or diploma above Bachelor's degree level	
Master's degree(s)	
Professional degree in pharmacy, dentistry, veterinary medicine, optometry, law, etc.	

Doctorate	

Baseline Mental and Physical Health (Objective 1)

10) ~~Considering physical health to include physical illness and injury, and mental health to include stress, depression, and emotional problems; for how many days in the past month was your: [AMS q15]~~
    - ~~Physical health **NOT** good (0‐31 days)~~
    - ~~Mental health **NOT** good (0‐31 days)~~

Physical Health

11) Do you have a regular medical doctor for your personal care? [CPHS q10]
    - Yes
    - No

12) In general, would you say your health is... ? [CCHS GEN_Q01]
    - Excellent
    - Very Good
    - Good
    - Fair
    - Poor

13) Compared to one year ago, how would you say your health is now? Is it...? [CCHS GEN_Q02A]
    - Much better now than one year ago
    - Somewhat better now than one year ago
    - About the same as one year ago
    - Somewhat worse now than one year ago
    - Much worse now than one year ago

14) How many hours do you usually spend sleeping each night? [CPHS Q21]
    - 0‐10, 11+

Exercise:

15) In an average week, how many times do you perform moderate, non‐exhausting exercises (e.g., fast walking, tennis, volleyball, dancing, easy swimming, or biking) [CPHS 33]
    - 0‐14+

16) On average, how long is each moderate exercise episode? [CPHS 34]
    - [blank] minutes/episode

17) In an average week, how many times do you perform strenuous exercises where your heart beats rapidly (e.g., jogging, soccer, aerobics, vigorous swimming or biking): [CPHS 35]
   - 0G14+

18) On average, how long is each strenuous exercise episode? [CPHS 36]
   - [blank] minutes/episode

<u>Nutrition:</u>

19) How often do you usually consume the following? Please mark any item you essentially never eat as "0" (mark one number and frequency for each item). [CPHS 38]

Example	Number of times							Per day	Per week	Per month
	0	1	2	3	4	5	6			
Fruit juice (e.g. orange, grapefruit, tomato)			X							X
Fruit, other than juice				X					X	

Example	Number of times							Per day	Per week	Per month
	0	1	2	3	4	5	6			
Fruit juice (e.g. orange, grapefruit, tomato)										
Fruit, other than juice										
Green salad										
Potatoes (not french fries, fried potatoes or potato chips)										
Carrots										
Servings of vegetables other than carrots, potatoes, or salad										
Caffeine (1 soda, 1 cup coffee, etc.)										
Vitamin/mineral supplement										

<u>Substance Use</u>

We would like to take this opportunity to remind you that your responses to all questions are <u>confidential</u>. No direct identifying data will be collected from participants. Demographic data with the potential for identifying individual participants will be collected anonymously (IP addresses will <u>not</u> be stored) and data will be kept confidential by the research investigators. All data, including demographic data, will only be presented at the aggregate level, with no identification of or reference to individual participants in published and presented reports.

Alcohol:

20) During the past month, on about how many days did you drink any alcoholic beverages? [AMS q27]
    - 0Ǧ31 days

21) On the days when you drank, about how many drinks did you drink, on average? (A drink is 1 can/bottle of beer or wine cooler, 1 glass of wine, 1 cocktail, or 1 shot of liquor). [AMS q28]
    - 0Ǧ9+

22) How often in the past month did you have ≥5 drinks on one occasion? [AMS q29]
    - 0Ǧ9+

Other Substances:

23) How often, if ever, have you used any of the drugs listed below? Do not include anything you used under a doctor's orders. [Modified from McCabe SE, et al. *Addiction* 2005]

	Never used	Used, but not in the past 12 months	Used, but not in the past 30 days	Used in the past 30 days
Stimulants: ie. Ritalin, Dexedrine, Adderall, Vyvanse				
Marijuana				

Smoking:

24) Have you smoked at least 100 cigarettes in your entire life? [AMS q30]
    - Yes
    - No➔Skip to Q29

25) Do you NOW smoke cigarettes every day, some days, or not at all? [AMS q31]
    - Every Day➔Skip to Q27
    - Some Days➔Skip to Q27
    - Not at all

26) How long has it been since you quit smoking cigarettes? [AMS q32]
    - ≤ 1 month
    - 2Ǧ5 months
    - 6Ǧ11 months
    - 1 year
    - 2Ǧ5 years

© 2021 Bahji A et al. *JAMA Network Open.*

– 6Ǥ11 years
– 12+ years

27) During the past 30 days, on the days you smoked, how many cigarettes did you smoke per day? [AMSq34]

28) On how many of the past 30 days did you: [AMS q33]
- Smoke part or all of a cigar
- Use any chewing tobacco, dip or snuff
- Smoke any tobacco in a pipe
- Smoke part or all of a cigarette

Mental Health

REMINDER [TO APPEAR ON ALL ONLINE SURVEY PAGES ASKING INFORMATION ABOUT MENTALHEALTH AND SUICIDE]:
We realize that some of the questions in the survey are personal in nature and deal with sensitive subject matter. Should you feel distress at any point during the survey, please feel free to discontinue your participation, and do not hesitate to visit http://suicideprevention.ca/thinkingCaboutCsuicide for information on a crisis centre in your province.

29) Maslach Burnout Inventory (MBI) G 2 item from Dyrbye et al., 2014; West et al., 2009

FOR MEDICAL STUDENTS WHO ARE IN THEIR SECOND YEAR OF STUDY OR LATER:
Following are 2 statements of jobCrelated feelings. For these questions consider medical school your "job" (attending lectures, clinical rotations, etc.). Please read each statement carefully and decide if you ever feel this way about your medical school training. If you never had this feeling, mark ''Never.'' If you have had this feeling, indicate how often you feel it by marking the response that best describes how frequently you felt that way. You may skip questions that you feel are inappropriate to your situation or you are unable to answer.

	Every day	*A few times a week*	*Once a week*	*A few times a month*	*Once a month or less*	*A few times a year or less*	*Never*
"I feel burned out from medical school"							
"I have become more							

callous toward people since I started medical school”							

FOR MEDICAL STUDENTS IN THEIR FIRST YEAR OF STUDY:

Following are 2 statements of jobCrelated feelings. For these questions think about your last year of working or school. Please read each statement carefully and decide if you ever feel this way about college or your last job. If you have never had this feeling, mark “Never”. If you have had this feeling, indicate how often you feel it by marking the response that best describes how frequently you felt that way. You may skip the questions that you feel are inappropriate to your situation or you are unable to answer.

	Every day	*A few times a week*	*Once a week*	*A few times a month*	*Once a month or less*	*A few times a year or less*	*Never*
“I feel burned out from schooling/work”							
“I have become more callous toward people since I have started schooling/work”							

30) The Connor Davidson Resilience Scale 2-item (CD-RISC2)

For each item, please indicate how much you agree with the following statements as they apply to you over the last month. If a particular situation has not occurred recently, answer according to how you think you would have felt.

	Not true at all *0*	*Rarely True* *1*	*Sometimes True* *2*	*Often True* *3*	*True nearly all of the time* *4*
I am able to adapt when changes occur.					
I tend to bounce back after illness, injury, or other hardships.					

<u>Mental Health Diagnoses</u>

For the following question regarding mood disorders, please only indicate longCterm conditions that are expected to last or have already lasted 6 months or more and that have been diagnosed by ahealth professional:

[This will appear at the top of the screen and will be visible for every question on mental health diagnoses]

Mood Disorders:

31) Do you have a mood disorder such as depression, bipolar disorder, mania or dysthymia? [CCHS CCC_280A]
    - Yes
    - No➔Skip to Q36

32) Since starting medical school, have you taken any time off of work or studies because of your mood disorder? [modified from Australia q35]
    - Yes
    - No

33) Since starting medical school, have you sought support or treatment for your mood disorder [Australian q43]
    - Yes
    - No➔Skip to Q36

34) From where did you seek personal support or professional treatment? (select all that apply) [Australian q44]
    - Friend
    - Family member
    - Spouse/partner
    - Fellow Student
    - University Services
    - Services provided specifically by your Faculty of Undergraduate Medicine (i.e. Learner Wellness/Student Affairs office)
    - Internet
    - Peer support program through a professional college or organization
    - CommunityGbased physician/psychiatrist/counsellor
    - Other personal support (please specify)

35) What type of treatment did you receive? (select all that apply): {Australian q45]
    - None
    - Counselling (e.g. cognitive behavioural therapy or interpersonal therapy)

- Medication
- Self‐help group (e.g. Alcoholics Anonymous)
- Alternative/complementary therapy
- Other treatment (please specify)

Anxiety Disorders:

36) Do you have an anxiety disorder such as a phobia, obsessive‐compulsive disorder or a panic disorder? [CCHS CCC_Q290A]
    - Yes
    - No➔Skip to Q41
37) Since starting medical school, have you taken any time off of work or studies because of anxiety? [Australia q42]
    - Yes
    - No

38) Since starting medical school, have you sought support or treatment for your anxiety disorder [Australian q43]
    - Yes
    - No➔Skip to Q41

39) From where did you seek personal support or professional treatment? (select all that apply) [Australian q44]
    - Friend
    - Family member
    - Spouse/partner
    - Fellow student
    - University Services
    - Services provided specifically by the Faculty of Undergraduate Medicine, i.e. Learner Wellness/Student Affairs office
    - Internet
    - Peer support program through a professional college or organization
    - Community‐based physician/psychiatrist/counsellor
    - Other personal support (please specify)

40) What type of treatment did you receive? (select all that apply): {Australian q45]
    - None
    - Counselling (e.g. cognitive behavioural therapy or interpersonal therapy)
    - Medication

– Self-help group
– Alternative/complementary therapy
– Other treatment (please specify)

In the past month, how often did you feel: [CCHS DIS_Q01A-J & CCHS PMH_Q01-14]

	All of the time	Most of the time	Some of the [illegible]	A little of the [illegible]	None of the time
41) ...tired out for no good reason?					
42) ...nervous?					
43) ...so nervous that nothing could calm you down?					
44) ...hopeless?					
45) ...restless or fidgety?					
46) ...so restless that you could not sit still?					
47) ...sad or depressed?					
48) ...so depressed that nothing could cheer you up?					
49) ...that everything was an effort?					
50) ...worthless?					
51) ...happy?					
52) ...interested in life?					
53) ...satisfied with your life?					
54) ...that you had something important to contribute to society?					
55) ...that you belonged to a community (like a social group, your neighbourhood, your city, your school)?					
56) **...that our society is becoming a better place for people like you?**					
57) ...that people are basically good?					
58) **...that the way our society works makes**					

2016-05-19 09:32:42

Mental Health Continuum [illegible]

2017-07-26 15:58:14

Kessler Psychological Distress

**sense to you?**					
59) **...that you liked most parts of your personality?**					
60) ..good at managing the responsibilities of your daily life?					
61) ...that you had warm and trusting relationships with others?					
62) ...that you had experiences that challenge you to grow and become a better person?					
63) ...confident to think or express your own ideas and opinions?					
64) ...that your life has a sense of direction or meaning to it?					

Suicide

65) Have you ever seriously thought about committing suicide or taking your own life? [CCHS SUI_Q02]

- Yes
- No

66) In the past 12 months, have you seriously thought about committing suicide or taking your own life? [CCHS SUI_Q03]

- Yes
- No

Functional Impact & Stigma (Objectives 2 & 4)

67) In the past 4 weeks, how often did your physical health make it difficult for you to handle your workload as a medical student? [CPHS q15]

- None of the time
- Some of the time
- Half of the time
- Most of the time
- All of the time

68) In the past 4 weeks, how often did your mental health make it difficult for you to handle your workload as a medical student? [CPHS q16]

- None of the time
- Some of the time
- Half of the time
- Most of the time
- All of the time

69) Please rate how strongly you agree with the following statements [Australia q65]:

	Strongly Agree	Agree	Neither Agree nor Disagree	Disagree	Strongly Disagree
Many doctors believe that a doctor with a history of depression or an anxiety disorder is less competent.					
Many doctors believe that experiencing depression or an anxiety disorder themselves is a sign of personal weakness.					
Doctors who experience depression or an anxiety disorder should change to a non‐clinical career.					
Doctors are less likely to appoint doctors with a history of depression or an anxiety disorder.					
Many doctors think less of doctors who have experienced depression or an anxiety disorder					
Doctors who have experienced depression or an anxiety disorder can achieve as much in their careers as those who have not.					
A doctor with a history of depression or an anxiety disorder is as reliable as the average doctor.					
Doctors feel they need to portray a healthy image.					
Doctors should be able to avoid depression or an anxiety disorder.					
Being a patient causes embarrassment for a doctor.					
Doctors tend to advise colleagues not to divulge a history of depression or an anxiety disorder.					
Doctors who experience depression or an anxiety disorder should be optimistic about their recovery.					

70) Please rate how strongly you agree with the following statements [AMS Q49‐61]

	Strongly Agree	Agree	Neither Agree nor Disagree	Disagree	Strongly Disagree
Overall, my medical school has encouraged me to lead a healthy life.					
My medical school tries to minimize student stress.					
My medical school encourages students' healthy eating.					
My classmates encourage each other to eat healthily.					
My medical school encourages students to exercise.					
My classmates encourage each other to exercise.					
My medical school discourages students from smoking.					
My classmates discourage each other from smoking.					
My medical school discourages students from binge drinking.					
My classmates discourage each other from binge drinking.					

71) If you re‐lived your life, would you still want to become a physician? [CPHS q3]

- Definitely
- Probably
- Maybe
- Probably not
- Definitely not

72) How much training have you had on talking to patients about (mark one for each): [AMS Q48]

	None	Some	Extensive
Nutrition			
Exercise/physical activity			
Smoking cessation (among smokers)			
Alcohol			
Mental health			

73) With a typical general medicine patient, how often do you actually talk about (mark one for each): [AMS Q47]

	Never/rarely	Sometimes	Usually/always	N/A
Nutrition				
Exercise/physical activity				
Smoking cessation (among smokers)				
Alcohol				
Mental health				

74) In your intended practice, how relevant do you think it will be to talk with your patients about the following topics (mark one for each): [reworded from AMS Q47]

	Not at all	Somewhat	Highly
Nutrition			
Exercise/physical activity			
Smoking cessation (among smokers)			
Alcohol			
Mental health			

Health Resources (Objective 3)

75) Whether or not you have been depressed or anxious or had substance use problems, where would you be comfortable seeking help from for these mental health problems? (please mark all that apply): [Australia q62]

Personal Support:

- Friend
- Family member
- Spouse/partner
- Fellow student
- University Services
- Services provided specifically by the Faculty of Undergraduate Medicine, i.e. Learner Wellness/Student Affairs office
- Internet
- Peer support program through a professional college or organization

- Other personal support (please specify)

Professional Support:

- General Practitioner
- Psychiatrist
- Psychologist/Counselor
- University Counseling/Psychological Services
- Faculty of Medicine‐specific counseling/psychological services
- Telephone helpline
- Other professional support (please specify)

76) Whether or not you have been depressed or anxious or had substance use problems, where would you **NOT** be comfortable seeking help from for these mental health problems? (please mark all that apply): [Australia q63]

Personal Support:

- Friend
- Family member
- Spouse/partner
- Fellow student
- University Services
- Services provided specifically by the Faculty of Undergraduate Medicine, i.e. Learner Wellness/Student Affairs office
- Internet
- Peer support program through a professional college or organization
- Other personal support (please specify)

Professional Support:

- General Practitioner
- Psychiatrist
- Psychologist/Counselor
- University Counseling/Psychological Services
- Faculty of Medicine‐specific Counseling/Psychological Services
- Telephone helpline
- Other professional support (please specify)

77) To the degree you would **NOT** be comfortable seeking help for depression, anxiety, or a substance use disorder, which of the following contribute to your hesitation (please mark all that apply) [Australia q64]:

- impact on licensure and right to practice
- concerns about career development/progression
- fear of lack of confidentiality/privacy

– impact on colleagues (i.e. letting fellow students down)
– lack of confidence in professional treatment
– reliance on self, do not want help
– difficulty recognizing symptoms of mental illness in yourself
– do not want to burden others
– do not believe it will help
– embarrassment
– lack of time
– fear of unwanted intervention
– cost
– stigmatizing attitudes to mental illness
– lack of knowledge about mental health services
– you are aware of the resources but they are inaccessible to you because of timing or location
– Other (specify)
– None of the above G I am comfortable seeking help

Learner Environment & Mistreatment (Objective 4)

78) During medical school I have been belittled by: [AMS q64]

	Never	Some	Severely
Students			
Residents/Fellows			
PreGclinical professors			
Clinical professors/attending physicians			
Patients			

79) During medical school I have been harassed by: [AMS q64]

	Never	Some	Severely
Students			
Residents/Fellows			
PreGclinical professors			
Clinical professors/attending physicians			
Patients			

80) What types of inappropriate behaviour have you personally experienced during medical school? Please select all that apply: [CAIR q77]

- Yelling, shaming, condescending
- Pressure to work long hours/do extra work
- Intimidation/bullying
- Sexual harassment
- Negative/unconstructive feedback
- Conflict/disrespect between specialties
- Senior staff unwilling to support/teach residents
- Staff gossip
- Racist, sexist, or homophobic remarks
- Other (please specify)
- None➔Skip to Q92

81) Referring to your answer to question 77, regarding personally experiencing inappropriate behavior in medical school, what steps did you take to address this behaviour? Please select as many as apply. [CAIR q78]

- Reported the incident to my course/clerkship director
- Reported the incident to the Undergraduate Medical Education Office
- Reported the incident to Student Affairs/Learner Affairs/Learner Wellness
- Talked to the individual about their behaviour
- Other (please specify)
- No steps taken

82) If no steps were taken, please specify the reasons why. Please select all that apply: [modified from CAIR q79]

- Did not feel the behaviour was sufficiently problematic
- Did not feel reporting the behaviour would change or remedy the situation
- Fear of reprisal from supervisors
- Fear of reprisal from peers
- Fear of reprisal from others
- Confidentiality concerns
- Unaware of reporting procedure
- Other (please specify)
- Unsure

Miscellaneous

83) (Optional) If you could name one thing that you feel would improve your and your colleagues' health and wellbeing, what would it be?

84) (Optional) How did you find the experience of taking this survey? Any other comments?

85) ***To show our appreciation to the other organizations that contribute to the mental health and wellness of Canadians, $2000 has been set aside for charitable contribution to Canadian organizations that work to advance mental health and wellness in Canada. For your participation, we would like your input on which organizations receive these funds. Please vote on which organization you would like us to donate to, and these funds will be distributed proportionally by percentage of votes.***

- **Canadian Mental Health Association**: www.cmha.ca (Promotes mental health and helps those with mental illness.)
- **Kids Help Phone**: org.kidshelpphone.ca (Canada's only 24/7 counselling and information service for young people, offers free and anonymous support to young people to improve their emotional health and well‐being.)
- **Breakfast Club of Canada**: www.breakfastclubcanada.org (Supports community‐based nutrition programs for children via awareness‐ and fund‐raising.)
- **Dreamcatcher Charitable Foundation**: www.dcfund.ca (Provides grants/funds in the areas of Arts and Culture, Education Support, Health Support, and Sports and Recreation to support First Nations communities and develop youth as future community leaders.)
- **Canadian Women's Foundation**: www.canadianwomen.org (Works to stop violence against women, end poverty, empower girls, and end human trafficking.)
- **Centre for Addiction and Mental Health:** (Canada's largest mental health and addiction teaching hospital, as well as one of the world's leading research centres in the area of addiction and mental health.)

REMINDER [TO APPEAR AGAIN AT THE END OF THE SURVEY]:

On behalf of the CFMS and FMEQ and the medical students of Canada who will benefit from your participation in this survey, thank you for your time and attention in this very important project!

We realize that some of the questions in the survey are personal in nature and deal with sensitive subject matter. Should you feel distress at any point during the survey, please feel free to discontinue your participation, and do not hesitate to visit ***http://suicideprevention.ca/thinking‐about‐suicide*** *for information on a crisis centre in your province.*

For more information on the range of physician health services offered to you by the CMA and Divisions, including access to confidential support services, please contact the physician health program in your

province/territory or the CMA Centre for Physician Health and Well4being at 148774CMA444YOU (148774 26244968). A full list of physician health programs and contact numbers is available at https://www.cma.ca/En/Pages/provincial4physician4health4programs.aspx

If you have questions about the survey, please contact the lead investigator, Brandon Maser, B.Sc., MD (Candidate), (bmaser@qmed.ca), or the UBC Research Subject Information Line at 604482248598.

AFTER EXITING THE SURVEY

"To show our appreciation for your participation in the CFMS4FMEQ National Health and Wellbeing Survey, we would like to provide you the option to enter into a draw for a chance to win one of 2 iPads. By clicking the link below, you will be taken to a completely separate questionnaire to enter your first & last name, and email address. Please note that your personal information will not be associated with your survey responses in any way. Your responses have already been submitted and we cannot link these with your contact info entered into the draw.

Entry into this draw is completely optional. If you have questions about the draw prize contest, please contact Brandon Maser, co4principal investigator, bmaser@qmed.ca

To enter your information, please copy and paste the following URL into your browser:
[Link to enter name and email address]"

[RESPONDENTS WILL HAVE THE OPTION CLICK ON A LINK AND BE TAKEN TO A SEPARATE SURVEYMONKEY SURVEY THAT IS NOT CONNECTED WITH THEIR RESPONSES. WE ARE NOT RECORDING IP ADDRESSES, NOR ARE WE TRACKING WHICH PARTICIPANTS CLICK ON THIS LINK, AS IT APPEARS AFTER SURVEY RESULTS ARE COMPLETELY SUBMITTED AND PARTICIPANTS HAVE EXITED THE SURVEY. THEREFORE, IT IS NOT POSSIBLE FOR US TO LINK RESPONDENTS' CONTACT INFORMATION WITH THEIR SURVEY RESPONSES]
